# Comprehensive Water Footprint of a University Campus in Colombia: Impact of Wastewater Treatment Modeling

**DOI:** 10.1007/s11270-022-05644-3

**Published:** 2022-05-13

**Authors:** Jose Luis Osorio-Tejada, Manuel Varón-Hoyos, Tito Morales-Pinzón

**Affiliations:** 1 Territorial Environmental Management Research Group –GAT, Universidad Tecnológica de Pereira, Pereira, Colombia; 2 School of Engineering, The University of Warwick, Coventry, UK

**Keywords:** Water footprint, Water accounting, Wastewater treatment, Life cycle assessment, Universities

## Abstract

Protection of water resources implies the responsible consumption, and the return of this resource with the best physicochemical conditions. In organizations, water is consumed both directly in their facilities and indirectly in the products or services acquired for their operation, requiring a water accounting based on the life cycle perspective. This study aims to assess the comprehensive water footprint of the main campus of the Technological University of Pereira (Colombia), based on the ISO 14046:2014 standard, and analyze the influence of wastewater treatment. Impacts on water scarcity were evaluated using the AWARE method, while the impacts on human health and ecosystems were evaluated using the ReCiPe method. Specific modeling of the wastewater treatment plants on campus was conducted. A total of 102,670 m^3^.y^−1^ of water scarcity was accounted for. Water consumption per person was 17.8 m^3^ of which 86.2% corresponded to indirect activities. Similarly, indirect activities were responsible for more than 98% of the impacts on human health and ecosystems, where more than 95% were due to infrastructure construction and 2% due to electricity consumption. Although the wastewater treatment on campus reduced the impact on ecosystems by 14%, if a tertiary treatment was added, these impacts would have a 40% of additional reduction. Efforts in recycling programs were also quantified in 712 m^3^ of avoided water scarcity for secondary users. The findings suggest focusing actions on sustainable construction and purchases to improve water management in organizations.

## Introduction

Water management as a natural resource has more and more tools, which allow obtaining more precise and detailed information on its consumption and degradation. Given that globalization has implied a growing connection between people and economies through trade (Fulton et al., 2014), it is necessary that the demand and supply of water coincide worldwide. Consequently, water has ceased to be a local resource to become a global resource (Hoekstra, 2013). Recently, methodological tools have been developed to optimize water resource management by evaluating its sustainability. In this sense, there are two indicators or approaches that have gained relevance in recent times. First, there is the so-called “Water Footprint,” which is the first methodology developed with the purpose of identifying and quantifying the environmental impacts related to water use (Fulton et al., 2014). The water footprint is an indicator defined as the amount of water consumed, both directly and indirectly along the supply chain of a good or service (Hoekstra et al., 2011). This indicator is a volumetric measure for both its consumption and contamination (Bai et al., 2018a; Mian et al., 2021). On the other hand, the methodology developed by the International Organization for Standardization (ISO) named “Environmental management—water footprint—principles, requirements and guidelines”—ISO 14046 (ISO, 2014) allows quantifying potential environmental impacts related to water use, based on the life cycle assessment (LCA) methodology (Bai et al., 2018a).

Both the water footprint as a volumetric indicator and the water footprint based on LCA have been used as tools to improve water management in economic activities such as textile production (Handayani et al., 2019; Li et al., 2021; Yang et al., 2020) food production (Bai et al., 2018b, 2021; Bazrafshan & Dehghanpir, 2020; Botti Abbade, 2020; Kashyap & Agarwal, 2020; Lee et al., 2018; Pascale Palhares et al., 2021; Ratchawat et al., 2020; Severo Santos & Pena Naval, 2020; West & Baxter, 2018; Zhai et al., 2021), construction (Kim et al., 2021; Mahdi Hosseinian & Mozhdeh Ghahari, 2021), energy production (Ansorge et al., 2020), mining (Chen et al., 2021; Yang et al., 2019), and tourism (Li, 2018).

Likewise, both methodologies have been used to assess the impacts of water use at the level of territorial entities (Li et al., 2020), hydrographic basins (Muratoglu, 2019), urban systems (Ruíz-Pérez et al., 2020), and technologies such as carbon capture and storage (Rosa et al., 2021). At the organization level, progress has been made that is reflected in the development of specific methodological proposals such as the Organizational Water Footprint (OWF) structured by Forin et al. (2020) which is based on the integration of aspects of ISO 14046 and ISO/TS 14,072 (Organizational LCA). Universities have begun to integrate the measurement of the water footprint in their plans and actions aimed at improving their environmental performance. In this sense, the Valaya Alongkorn Rajabhat University (Thailand) (Kandananond, 2019) calculated the water footprint of the production of fuels and electricity consumed in the institution, and the Kathmandu University (Nepal) (Vaidya et al., 2021) evaluated the interaction between food, energy, and water. In both cases, these studies were conducted based on the water footprint method proposed by Hoekstra et al. (2011), in which the water use can be analyzed independently by concepts of blue water footprint (surface and groundwater consumption), green water footprint (rainwater consumption), and the gray water footprint (pollution of freshwater).

In Latin America, water footprint studies have been conducted for the agricultural sector, the mining sector, the chemical industry, the food, and beverage processing and transformation industry, the cement industry, and floriculture (CADIS, 2016). Regarding water footprint studies in higher education institutions in Latin America, the following institutions have hitherto reported their results: the CUCEA Study Center of the University of Guadalajara (Mexico) (Agua y Ciudad & Consultoría y Proyectos SC, 2016), the Pontificia Universidad Católica de Chile (Pontificia Universidad Católica de Chile, 2020), and at the National University of Costa Rica (Chavarría et al., 2020). For its part, in Colombia, the water footprint has been quantified in institutions such as the Catholic University of Manizales (Loaiza & Quiceno, 2018), the University of Córdoba (Montería Campus) (Contreras & Torres, 2017), the Autonomous University of the West (Urrutia, 2019), and the Santo Tomás University (Aguas Claras Headquarters) (Bonilla, 2020). However, these water footprint studies have not applied the four phases methodology described by the ISO 14046 standard, accounting only for water consumption, without including the impacts of water degradation, such as eutrophication and toxicity.

As part of the advances in the implementation of its institutional environmental policy, in the present study, the Technological University of Pereira (UTP) as an official Colombian higher education institution aims to present the results of the measurement of the comprehensive water footprint for the evaluation of the direct and indirect impacts of its activities on water resources. This measurement was made with the ISO 14046 standard as a methodological reference, which makes this study one of the first comprehensive water footprint assessments in Colombian universities.

In addition to measuring the direct and indirect impacts of the UTP’s institutional activities on water resources, inventories of the drinking water used and the specific treatment of wastewater within the campus were modeled, using a calculation tool developed in the framework of this project. This additional contribution to the research was necessary given that the university has two treatment plants in order to return the water used in acceptable conditions to the basin, through treatments and scales different from those modeled in the databases for life cycle inventory analysis.

## Methods

The international standard ISO 14046 (ISO, 2014) for water footprint assessments is based on the international standards ISO 14040 (ISO, 2006a) and ISO 14044 (ISO, 2006b) for LCA studies. The objective of this approach is to quantify the potential impacts related to the use of water in the life cycle of a product, service, or organization, considering impacts on ecosystems, human health, and resources.

**Fig. 1:**
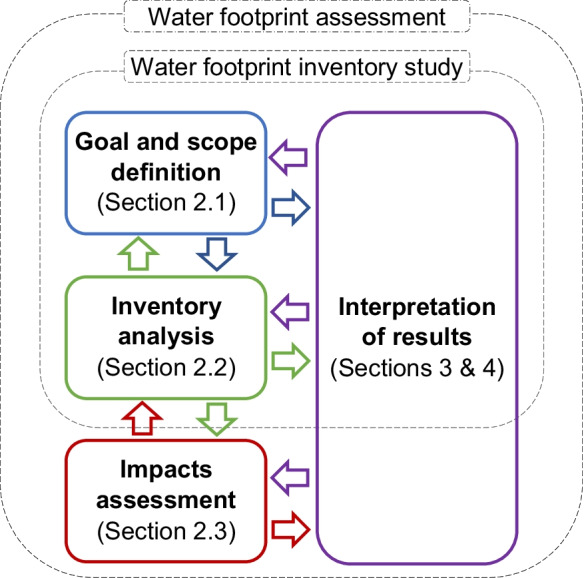
Phases of a water footprint assessment. Adapted from ISO 14046 (ISO, 2,014)

The water footprint assessment has four iterative phases (see Fig. 1): goal and scope definition, inventory analysis, impacts assessment, and interpretation. It can be part of an LCA study or be an independent assessment.

In contrast to LCA studies, the ISO 14046 standard presents the possibility of excluding the impacts assessment phase when the objective is only the quantification of water consumption in a water footprint inventory study. Yet, in our study for the comprehensive water footprint assessment of the university campus, we considered the four phases detailed in Fig. 1.

### Goal and Scope Definition

The objective of this study was to assess the main environmental aspects associated with water usage on the main campus of the UTP and analyze the influence of wastewater treatment and other water management strategies in this regard.

The functional unit was defined as the set of impacts generated on the water resource by the teaching, research, and extension activities carried out in 2017 on the main UTP campus, located in Los Álamos, southeast of Pereira, Colombia. Regarding the reference flows in 2017, the UTP awarded degrees to 2652 undergraduate students and 639 graduate students, published 174 scientific articles in peer-reviewed journals, and registered 20 patents (UTP, 2018).

The year 2017 was defined as a base year because in 2018, there was a strike of national university students in Colombia, which affected the normal development of activities in the academic periods 2018 and 2019, while the COVID-19 restrictions affected activities in 2020 and until the submission date of the present manuscript.

Regarding the scope of the study, the quantification of the water footprint of the UTP as an organization was defined. Consequently, the impacts of both the activities directly controlled by the university (direct impacts) and the activities associated with the institutional work that are not controlled by the institution (indirect impacts) were evaluated (Table 1).

**Fig. 2:**
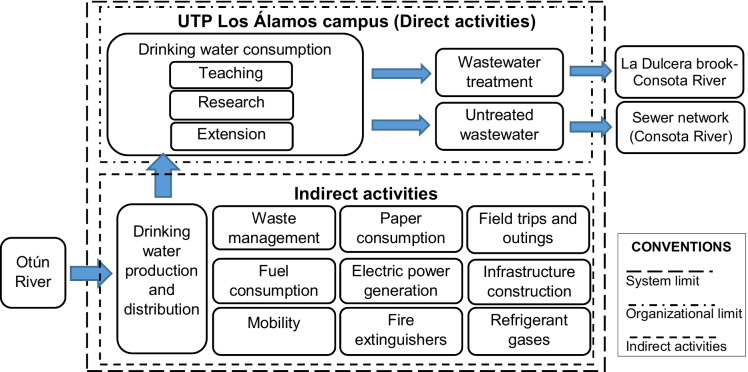
System boundaries

In this sense, the main UTP campus was established as the organizational limit (Fig. 2). The control approach was used to consolidate the environmental impacts because the UTP has control over the campus, its physical plant, and the activities conducted within its geographical limits.

### Inventory Analysis

Most of the collected data was based on primary sources provided by the different university departments such as the Institutional Services division, the Water and Sanitation Research group (GIAS), General Warehouse, Environmental Management Center, Administrative and Financial Vice-rectory, and the Office of Planning.

Based on the results of the characterization of the wastewater, both influent and effluent of each wastewater treatment plant (WWTP) of the campus, provided by the GIAS group, the emissions to air, water, and soil, were estimated. This inventory of emissions generated not only by WWTP but also by the post-treatment of the sludge resulting from the plant was created by own modeling, presented later in Sect. 2.2.1.

Information related to daily commuting was collected based on a survey developed by the authors and applied to 1287 students, 210 professors, and 70 administrative employees (Varón-Hoyos et al., 2021). From this survey, an origin–destination matrix was developed with the distances traveled from homes to campus and the type of vehicle in specific public or private services (size, manufacturer, Euro standard, and type of fuel).

The data relating to the indirect activities that UTP generates on the water were obtained from the reference values available in the Ecoinvent 3.6 database, using the SimaPro 9 software, with the allocation at the point of substitution, unit (APOS, U) approach. For inputs or activities for which there was no specific dataset for Colombia, new specific inventories were created from global inventories, adapting the specific energy flows and transportation, considering fuels used in the region with 10% biofuel of national origin. Likewise, a specific inventory was drawn up for the drinking water because the generic and specific inventories for Colombia considered a mix of different extraction sources such as wells, rivers, and lakes, and various purification techniques, different from the one used in the municipal water company of Pereira, which extracts all the water from the Otún river. The detailed activity data and the lifecycle inventories datasets per activity are presented in Table 4 from the appendix.

The material and energy balances of the treatment of non-hazardous solid waste for reuse and recycling purposes, as well as the water savings due to the use of the resulting materials in secondary products, were also inventoried. However, the impacts of these secondary use were not accounted for in the total water footprint of the campus because these impacts are partially allocated to third-party organizations where the resulting materials are used.

#### Wastewater Treatment Inventory Model

Since the city does not have a municipal wastewater treatment service, the university has two treatment plants to return the used water in acceptable conditions to the basin, using primary and secondary treatments. In this sense, the inventories available in databases for WWTP did not apply to this study because in these databases for life cycle inventories such as Ecoinvent (ETH, 2020), the modeled plants are at a municipal scale for the treatment of average domestic wastewater and using tertiary treatment technologies, without the possibility of modifying these parameters in SimaPro.

Therefore, within the framework of this study, it was necessary to develop a tool for estimating emissions generated by WWTP and by water discharged into rivers with and without treatment. This model development was necessary because no applicable tool was found for this study. Corominas et al. (2020) identified 10 tools for the analysis of environmental impacts of WWTP based on LCA. Among these tools, most of them focus on the estimation of greenhouse gas emissions to air and the emissions of pollutants to water. Some of these tools only calculate these emissions based on the type of WWTP and the amount of treated water, making the estimates with fixed average influent parameters, while other tools also include emissions to air, water, and soil generated by the consumption of energy and materials used in the construction of the WWTP. However, the main limitation of these tools is that they do not allow modifying the physicochemical composition of the water to be treated to estimate the emissions generated in the plant, the specific composition of the effluent, and the emissions to air, water, and soil, generated by the residual sludge from the treatment process.

This is essential when looking to analyze the potential impact of specific wastewater treatment in industries by the composition of wastewater in each type of production process. The Ecoinvent database includes inventories for domestic and industrial wastewater treatment for samples generated in Switzerland (Doka, 2009), generating high uncertainty when these inventories are used to model WWTP from other non-European countries. In addition to this, these emission inventories are applicable for three-phase municipal WWTP (with tertiary treatment), much larger than the two WWTP used in the university, which only have secondary treatment systems.

In order to address these limitations, models such as SewageLCI have been developed (Birkved & Dijkman, 2012). This model provides a mass balance of chemicals that are released to the wastewater system, as well as all the remaining fractions, such as the fraction released to air, surface water, and the fraction transferred to sludge. However, the SewageLCI model does not include inventories for infrastructure, energy consumption, and other materials used during treatment. To fill this gap, Muñoz et al. (2017) developed WW LCI 1.0, also including sludge treatment and removal. A limitation of this model is that it only considers wastewater treatment at the secondary treatment level. For this reason, Kalbar et al. (2018) developed the WW LCI 2.0 model, which integrates the analysis of tertiary treatment in WWTP, mainly dedicated to reducing phosphates in wastewater. However, its operation requires a trained professional given its complexity, which makes it unlikely that this tool can be used by small companies.

**Fig. 3:**
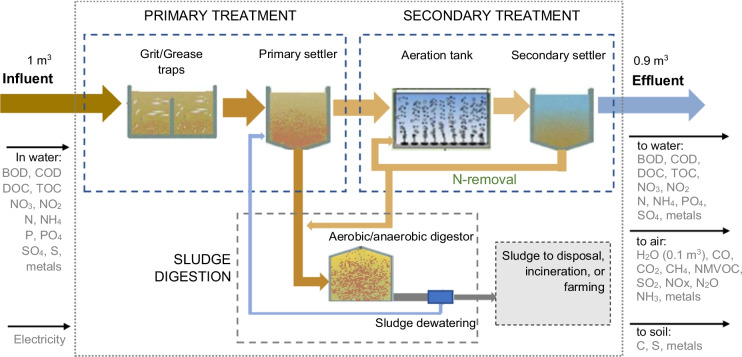
Emission flows in WWTPs with secondary treatment

Therefore, a simplified model was developed for this study, which, based on the characterization of the water influent of the WWTP, estimates the balance of materials and emissions in the treatment plants and in the post-treatment of the resulting sludge. This model also allows selecting the fraction of sludge that goes to incineration, sanitary landfill, or to agricultural soils (Fig. 3). For this specific case of the UTP, the whole resulting sludge is dedicated to the improvement of agricultural soils.

The model is based on transfer coefficients for each compound in each stage of the treatment process. That is, in this case, 32% of the total organic carbon is retained in the primary settler sludge, 33.8% in the secondary settler sludge, 24.5% is oxidized and emitted into the air mostly in the form of CO_2_, and the remaining 9.7% continues in the effluent (Doka, 2009). Likewise, transfer coefficients are applied at each stage for nitrogen, phosphorus, sulfur compounds, and other metallic and non-metallic elements present in the wastewater to be treated (Boller & Hafliger, 1996; Doka, 2009; Koppe & Stotzek, 1993). The detailed transfer coefficients and data used in the inventories modeling are presented in the appendix in Tables 5, 6, 7, and 8.

For the specific inventories of the two WWTP of the campus, the flows involved in their construction and the energy costs of their daily operation were not included because these are already included in the items of electricity consumption and infrastructure construction of the campus.

### Impact Assessment

This study evaluated the direct and indirect impact on the water resource of the activities carried out in the UTP in the base year 2017 by performing two types of analysis: the measurement of the water footprint due to scarcity and the approximate measurement of the comprehensive water footprint. These analyzes were conducted for both direct and indirect uses of water.

#### Water Footprint—Scarcity

To establish the water footprint due to scarcity generated by both direct and indirect activities, the indicator Available WAter REmaining (AWARE) (Boulay et al., 2015) was used. AWARE is a midpoint method developed to assess the remaining water available by area in a basin, once the water needs for humans and aquatic ecosystems have been met (Puerto & Gmünder, 2017). The water scarcity value is obtained from the multiplication of water consumption (m^3^) by a regional water stress factor (availability and demand). AWARE was chosen for this study over other methods for the water scarcity assessment, such as the Water Stress Index (Pfister et al., 2009) and the Blue Water Scarcity ratio (Hoekstra et al., 2011) because the water stress factors for the AWARE method were adapted at sub-basin level in Colombia annual data from the IDEAM National Water Studies in 2014 (CADIS, 2016). In this sense, the AWARE method offers more accurate estimates for site-specific studies in Colombia than other methods based on average global or national characterization factors.

The water consumption was obtained from the life cycle impact assessment method ReCiPe 2016 (Huijbregts et al., 2017), while the regional water stress factors by hydrographic subzones of Colombia were obtained from the AWARE layer for Google Earth, from the National Water Study (IDEAM, 2015).

#### Water Footprint—Degradation

For the comprehensive water footprint assessment of the UTP campus, four midpoint impact categories were included: freshwater eutrophication, marine eutrophication, freshwater ecotoxicity, and marine ecotoxicity. These impact categories were selected based on the recommendations for this kind of study in Latin America (CADIS, 2016), in which besides the relevance of analyzing toxicity impacts, the analysis of the water eutrophication has become relevant in regions with high agricultural activity given the use of fertilizers. These impact categories are also relevant in the evaluated case study given the specific water treatment on campus in which nitrogen compounds are reduced, but phosphorus compounds are entirely discharged into rivers.

**Fig. 4:**
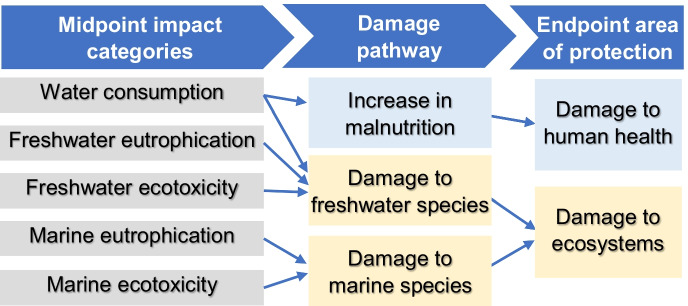
Relationship between impact categories and protection areas

For this impacts assessment, the ReCiPe 2016 method was chosen (Huijbregts et al., 2017) because, among the most updated methods, it includes the selected five midpoint categories, as well as provides their aggregation into endpoint categories. This method transforms the life cycle inventory data into the midpoint impact categories, which represent the environmental impact per unit of stressor (for example, per kilogram of resource used or emission released) (Huijbregts et al., 2017). Midpoint categories represent impacts that occur due to impact pathways or flows, while the endpoint categories refer to potential impacts of the analyzed midpoint categories in protection areas, such as human health and ecosystem quality, generated by a reduction in the availability or quality of water (Fig. 4).

Impacts on human health are expressed in DALYs *(Disability Adjusted Life Years)* and represent the number of years lost due to illness or premature death due to lack of water, either for food production or for direct consumption of drinking water. The impacts on the quality of ecosystems are expressed in species.year or PDF.year *(Potentially Disappeared Fraction of species)*, which represent the fraction of species that disappeared in a year.

## Results

### Uses of Water at the UTP Campus

#### Direct Uses of Water

##### Water Inlets

In 2017, a total of 48,239 m^3^ of drinking water from the purification plant operated by *Aguas y Aguas de Pereira* were used on the UTP campus. All the purified water came from the Otún River; that is, from a surface water source (Varón-Hoyos et al., 2019). This drinking water was used for sanitary facilities, laboratories, cafeterias, and kiosks, as well as for cleaning classrooms, offices, and common areas. Drinking water was also used to irrigate green areas.

##### Water Outlets

In 2017, a total of 18,523.8 m^3^ of drinking water were used to irrigate gardens and other green areas of the campus, which represented 38.4% of the water consumed. On the other hand, 10,408 m^3^ of untreated wastewater was discharged from the campus to the municipal sewer network, corresponding to 21.6% of the volume of water consumed on campus. Likewise, 911.4 m^3^ of water resulting from the treatment process conducted at the WWTP plant located in the vicinity of the Faculty of Fine Arts (Fine Arts WWTP) was discharged into the Consota river, which represented 1.9% of the total water consumed. While 16,447 m^3^ of water from the WWTP located in the vicinity of the campus sports facilities (Sports WWTP) were discharged into La Dulcera brook, which corresponded to 34.1% of the total water consumed. Finally, the output of 1928.6 m^3^ of water (4% of the total water entering the campus) was due to evaporation during the operation of the two wastewater treatment plants, Table 9.

Regarding the composition of the wastewater discharged without treatment, it was assumed to have similar characteristics to those of the influent of the Fine Arts WWTP presented in Table 5 because it corresponds to nearby buildings. For each considered effluent, the estimated emission inventories to air, water, and soil are presented in the appendix in Tables 10, 11, and 12, respectively. Note that for these inventories, the whole sludge generated in the wastewater treatment processes on the UTP campus is used for farming purposes. Therefore, since no sludge is incinerated, there is no slag residue going to landfills, then no emissions would be transferred to groundwater.

#### Indirect Uses of Water

The indirect use of water in the UTP is related to activities needed for the development of teaching, research, and extension work. These activities were the electricity consumption, the gases recharging of fire extinguishers and refrigerants of air conditioners, the consumption of fuels both in fixed sources and in mobile sources, transport of employees in official trips, transport for field trips of students, mobility towards the campus of employees and students, paper consumption, infrastructure construction, and the management of solid waste both ordinary and dangerous. The total water use due to indirect activities was 309,732.7 m^3^. The detailed consumption of each activity is presented in Table 13.

### Water Footprint Impacts Assessment

#### Scarcity Impact

Since the water used in the UTP does not return to the same Otún river basin from where it is initially extracted by *Aguas y Aguas de Pereira*, this effluent from the campus is also considered consumed water, according to the guidelines of the ISO 14046 standard. In this sense, the total consumed water in 2017 was 48,239 m^3^, of which 38.4% evaporated or infiltrated the soil during irrigation activities, 4% evaporated during wastewater treatment, and the remaining 57.6% returned to the Consota River, whether treated or untreated. The indirect water consumption was 309,715 m^3^, representing 86.5% of the total 357,972 m^3^.

**Fig. 5:**
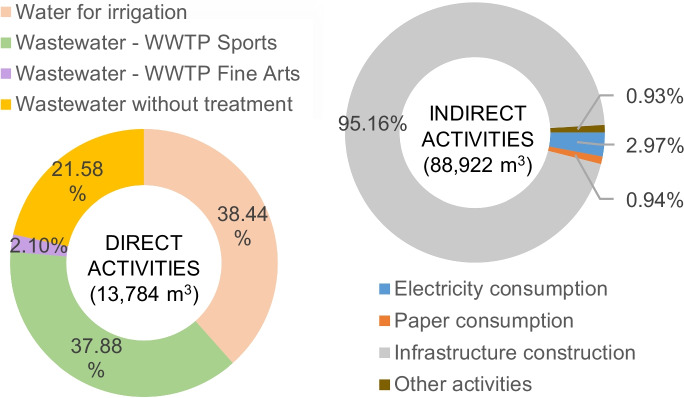
Contributions of direct and indirect activities on scarcity (AWARE)

Evaluating the impact due to scarcity using the AWARE indicator, the total water scarcity indicator for 2017 was 102,670.3 m^3^, of which 13,748 m^3^ (13.4%) were due to direct impacts and 88,922 m^3^ (86.6%) were due to indirect impacts. Most of the direct impact on the scarcity was due to the consumption of water for irrigation of green areas and gardens (38.4%) and due to the effluent from the Sports WWTP (37.9%). On the other hand, the indirect impact was mainly due to the infrastructure construction (95.2%) and electricity consumption (3%) (Fig. 5).

#### Degradation Impact

**Fig. 6:**
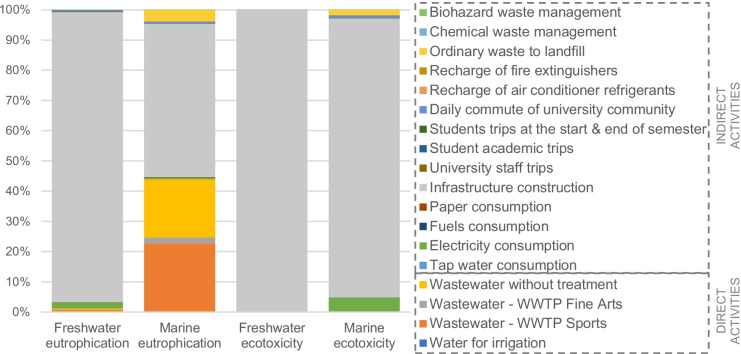
Impacts on water quality

In addition to the impacts related to the degradation of water due to the production of the inputs used on campus (indirect impact), the return of wastewater to the environment and the previous treatment process of this wastewater and the resulting sludge also generate impacts in water quality (direct impact). Most of the impacts on water consumed in 2017 were due to indirect activities, mainly the infrastructure construction (Fig. 6). Electricity consumption was the second responsible for the water ecotoxicity mainly due to copper particles and other toxic elements used in the transmission and distribution networks. The total impacts in the evaluated year on freshwater eutrophication, marine eutrophication, freshwater ecotoxicity, and marine ecotoxicity were 8.3 kg P-eq, 1.6 kg N-eq, 130.2 × 10^6^ kg 1,4DCB-eq, and 2.42 × 10^6^ 1,4DCB-eq. The detailed shares of each activity are presented in Table 14. In general, indirect activities contribute to 100% of the ecotoxicity impacts and to 98.8 and 56.1% of the eutrophication impacts on fresh and marine water, respectively.

In relation to the water consumption, the eutrophication impacts on freshwater by direct and indirect activities are about 2.07 × 10^–6^ kg P-eq/m^3^ and 2.65 × 10^–5^ kg P-eq/m^3^, respectively. This demonstrates that academic activities in the UTP generate fewer negative impacts on freshwater than other indirect activities per each m^3^ of water consumed.

Regarding the final damages related to water consumption and contamination, the campus generated a total impact on human health of 0.3 DALYs. Given that the damage to human health in this study is related to water consumption (see Fig. 4), the contributions of each activity are similar to the results of the impact due to scarcity in Fig. 5.

**Fig. 7:**
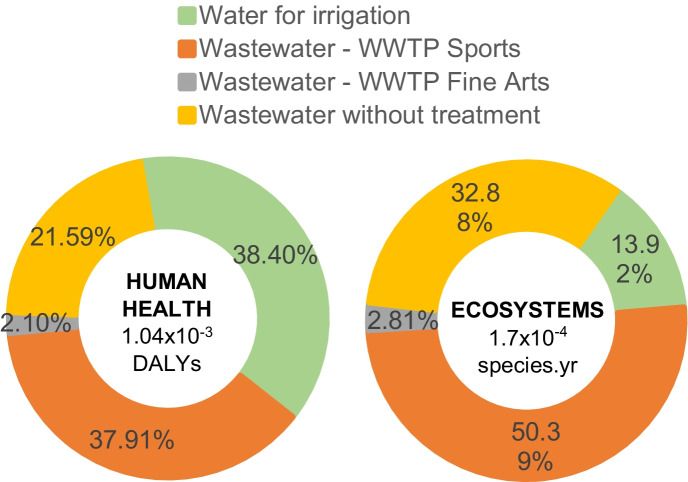
Contributions of direct activities to final damages

Of the final damages to ecosystems, equivalent to a total of 7.6 × 10^–3^ species per year, 1.4% corresponded to direct activities. The reduction of species in ecosystems is also caused by water scarcity, but water pollution by eutrophication and ecotoxicity have a more relevant impact. Hence, the consumption of water in irrigation activities has a lower impact on ecosystems than the wastewater discharged to basins (Fig. 7).

**Fig. 8:**
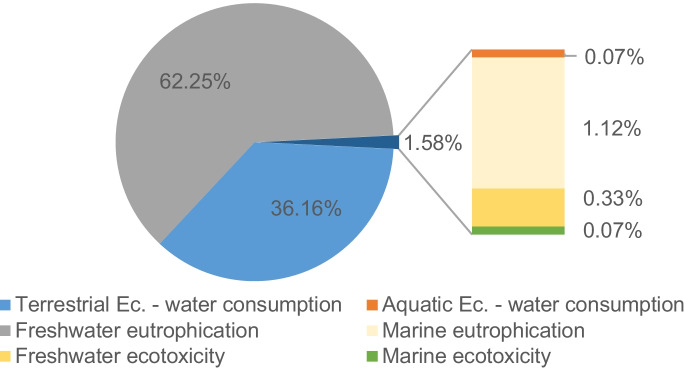
Contribution of impact categories in damage to ecosystems (Ec) by direct activities

It is observed that, despite being treated, the effluent from the WWTP generates most of the damage to the ecosystems. This is because of the total damages generated by direct impacts on ecosystems, equivalent to 1.1 × 10^–4^ species per year, 62.3% correspond to the category of freshwater eutrophication (Fig. 8), caused by 100.1 kg of P Eq. (1.2% of the total of this category, Table 14).

In this sense, given that the two WWTPs on campus do not reduce phosphorus in the water, each m^3^ of effluent has the same freshwater eutrophication potential as the effluent without treatment. This explains that the Sports WWTP has a greater impact on ecosystems in Fig. 8 due to the higher quantity of water processed in this plant.

### Impact of Waste Recycling

**Fig. 9:**
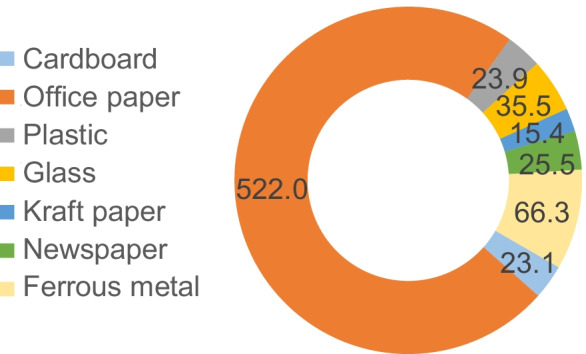
Scarcity (m^3^) avoided by recycling waste in 2017

Given that the water savings due to the recycling of non-hazardous waste would be assigned to the organization that uses the resulting materials in other products, these results are presented separately in Fig. 9.

The recycling of a total of 35,758 kg of ordinary waste on campus in 2017 might reduce the scarcity of 712 m^3^ of water due to the lower need for extraction and production of equivalent virgin material for the manufacture of third-party products. From this total, 522 m^3^ of water was due to the recycling of office paper.

## Discussion

By 2017, the campus had 18,839 students, 1327 teachers, and 399 administrative officials, which indicates that the total water footprint was 17.8 m^3^ per member of the university community. Other universities that have published this indicator, such as the Kathmandu University (Nepal) (Vaidya et al., 2021), reported a figure of 187.3 m^3^ per capita, including the water consumed in the student residences. However, since this and other reports from higher education institutions did not use the ISO 14046 methodology or consider similar activities, it was not possible to analyze whether the obtained indicator per capita is high or low. In this sense, the analysis of the obtained results in this study focuses on the methodological aspects used through sensitivity analysis.

In this study, inventories for the specific wastewater treatments within the campus were modeled, rather than using generic inventories. Table 2 shows the variation in results due to direct activities if the specific modeling of the WWTP had not been conducted. In other words, if all the wastewater generated by the campus was treated with the average treatment process for a plant with a capacity of 1.6 × 10^8^ L per year (Class 5), which is the smallest available in Ecoinvent 3.6. It is observed in Table 2 that the impacts on human health, related to water consumption, increased when using the generic wastewater treatment inventory. This is because the Ecoinvent inventory includes the impacts of the materials and supplies used in the construction of the WWTP and the chemical inputs for tertiary treatment to reduce phosphates, which were not considered in the specific modeling. Yet, impacts on ecosystems are reduced by 36.3% because the tertiary treatment greatly reduces freshwater eutrophication, which is the main responsible for the total damage to species per year caused by direct activities. These significant variations in the results confirm the relevance of the modeling of specific inventories for these WWTP to achieve representative results for the campus in the evaluated period.

Nevertheless, the utilization of the dataset for WWTP with tertiary treatment is not an option to consider in this case because the WWTPs on the UTP campus have up to secondary treatment. In this sense, if practitioners wrongly selected a WWTP with a different function from the one used, the estimated impacts would have great variations of over 30%. Therefore, it is demonstrated the need of conducting specific modeling for the inventories creation of wastewater treatment processes performed inside organizations. The model described in this work can be used by any organization using WWTP with secondary treatment when the wastewater influent or effluent composition is known. Moreover, if the wastewater composition is unknown, other higher education institutions in Latin America could use the estimated emissions inventories presented in this work for the two WWTPs or the effluent without treatment, instead of using datasets with average residential wastewater composition from other continents. Note that the pollutant emissions from the Fine Arts WWTP were higher than those from the Sports WWTP because the latter was updated with more modern equipment. After the finalization of this work the Fine Arts WWTP was also improved, then it is expected that both WWTPs have similar performance.

To analyze the importance of wastewater treatment, a second sensitivity analysis was conducted to observe to what extent the decision to return all the wastewater without any treatment to the municipal sewer would affect the final impacts (Table 3). It is observed that there was no difference in the impacts on human health due to water consumption because the evaporated water in the WWTP and the effluent are considered as consumed water for being discharged into a different water basin. On the other hand, the damage to ecosystems would increase by around 14% due to the higher content of organic material, nitrogen compounds, and heavy metals that would directly reach the environment. Although, since only 40% of the total wastewater was treated, the final impact on ecosystems would increase in total by 7.7% if it were not treated.

Regarding the quality of the results, in general, a medium level of uncertainty is considered. In the first place, most of the activity data present high precision since they are values measured directly by the university, except for the amounts of refrigerant gases and recharged extinguishing agents, which had to be estimated. However, these activity data represent a low percentage of the total impacts on the water footprint; therefore, the general reliability of the results is not affected. Second, most of the inputs and process inventories in the database are not created specifically for Colombia. However, these global or existing inventories for other countries were adapted to the case studied to make them more representative and thus reduce their uncertainty. And, thirdly, although the inventory modeling of the wastewater treatment process was conducted based on transfer coefficients also used by Ecoinvent, the wastewater composition data corresponded to the average of samples collected for 2 days, thus being unrepresentative for the 365 days of a year. For this last reason, we consider that the results for the impacts of direct activities on the water footprint might have high uncertainty.

Therefore, it is important to mention that the optimization of the internal information systems of the university, the availability of specific wastewater treatment inventories for Colombia, and the constant monitoring of the activity of the WWTPs are factors that can help to conduct this kind of studies with more appropriate levels of uncertainty.

On the other hand, considering that the indirect uses of water were the cause of most of the impacts, both in terms of scarcity and quality of the water resource, it is pertinent to point out the need for the UTP to implement measures that promote the sustainable construction and the use of materials that have a smaller water footprint than those that are usually used. Likewise, it is proposed to promote energy-saving and efficiency, and sustainable mobility by students, lecturers, and administrative staff.

## Conclusions

The comprehensive assessment of the water footprint of the main campus of the UTP was conducted based on the ISO 14046 standard with the aim of analyzing the main environmental aspects associated with the direct and indirect use of water and the influence of wastewater treatment processes in this regard.

The impact assessment was conducted by calculating the water footprint due to scarcity with the AWARE method and the impacts of water degradation on human health and ecosystems using the ReCiPe 2016 method. The water consumption per member of the university community was 17.8 m^3^ of which 86.5% corresponded to indirect activities. The water scarcity impact through the AWARE indicator for the evaluated year was a total of 102,670 m^3^. This study demonstrated that academic activities generate insignificant ecotoxicity impacts and 10 times less freshwater eutrophication than indirect activities per each m^3^ of water consumed. This study allowed us to identify the critical points that generate potential impacts associated with water scarcity and degradation, such as wastewater treatment, and indirect activities such as infrastructure construction, electricity consumption, and the daily commuting of the university community.

Specific modeling of the WWTPs located inside the campus had to be conducted because the generic datasets for municipal WWTP did not represent the water compositions and treatment methods used in the UTP plants. This novel simplified model for the inventory analysis of wastewater treatment allowed obtaining greater precision in the results and reduced uncertainty. Wastewater treatment on campus reduced about 8% of the impact on ecosystems. If a tertiary treatment were added to reduce phosphates, the discharged water after treatment would have an additional impacts reduction on ecosystems of around 40%.

Efforts in recycling programs were also quantified in 712 m^3^ of avoided water scarcity by recycling 35 tons of ordinary waste in the evaluated year. This recycling of ordinary waste also contributes to reducing the impacts of the transportation and disposal of this waste in landfills.

Regarding the impacts of direct activities on the water footprint, it is suggested to rationalize the use of water for the gardens and green areas of the campus, as well as to reduce the amount of wastewater discharged into the sewage system without prior treatment, since this discharge significantly increases the direct impact on ecosystems. In the case of the impacts of indirect activities, it is necessary to promote sustainable construction. Moreover, it is necessary to reduce the consumption of grid electricity because this input is the second responsible for the scarcity and ecotoxicity of water, the latter mainly due to copper particles and other toxic elements used in the transmission and distribution networks. Hence, the use of energy produced locally from renewable sources can improve these indicators.

In general, in addition to being the first analysis of the comprehensive water footprint of a higher education institution, this study serves as the basis for defining strategies for the optimization of internal processes and sustainable purchases that allow progress in water management in organizations.

**Table 1:** Activities included in this water footprint assessment

	Activity data
Direct	Drinking water consumption
	Wastewater treatment
Indirect	Purification and distribution of water
	Fire extinguishers (agent recharge)
	Air conditioners (refrigerant gases)
	Fuels (fixed and mobile sources)
	Transport for staff trips
	Transport for student field trips
	Daily mobility to the campus
	Students transport hometown-Pereira
	Electricity consumption
	Paper consumption
	Infrastructure construction
	Ordinary waste management
	Hazardous waste management

**Table 2:** Specific vs generic wastewater treatment sensitivity analysis

Input or activity	Variation in final damages	
	Human health	Ecosystems
Water for irrigation	0.0%	0.0%
Sports WWTP	+ 48.9%	− 39.2%
Fine Arts WWTP	+ 48.9%	− 39.6%
Untreated wastewater	+ 48.9%	− 47%
Direct impact	** + 30.1%**	− **36.3%**

**Table 3:** Sensitivity analysis without wastewater treatment

Input or activity	Variation in final damages	
	Human health	Ecosystems
Water for irrigation	0.0%	0.0%
Sports WWTP	0.0%	+ 14.6%
Fine Arts WWTP	0.0%	+ 13.9%
Untreated wastewater	0.0%	0.0%
Direct impact	**0.0%**	+ 7.7%

## Data Availability

The datasets generated during and/or analyzed during the current study are available from the corresponding author on reasonable request.

## Appendices

**Table 4:** Activity data and lifecycle inventories datasets

Input or activity (unit)		Total quantity	Remarks	Ecoinvent dataset
Drinking water (m^3^)		48,239	From the municipal water purification plant	Tap water {Pereira}| production, conventional treatment, and distribution
Electricity (kWh)		2,544,020	National grid electricity	Electricity, low voltage {CO}| market for electricity, low voltage
Fuels	Diesel B10 (gallon)	2624.5	Diesel B10: 1,469.4 gallons were consumed in stationary sources and 1,155.1 gallons were consumed in mobile sources	Diesel B10 {CO}| market for diesel
	Gasoline E10 (gallon)	704.1		Gasoline E10 {CO}| market for petrol, unleaded
	Natural gas (m^3^)	11,830		Natural gas, low pressure {CO}| market
Paper (kg)	White office paper	10,107.1	White office paper: 7,055.5 kg were consumed in offices and 3,051.6 kg were consumed in photocopies kiosks	Paper, woodfree, uncoated {CO}| market
	Newsprint	1890		Paper, newsprint virgin {CO}| market
	Cardboard	326.1		Containerboard, linerboard {CO}| market for kraft liner
Infrastructure construction (m^2^)		86,773		Building, hall {CO}| market
Staff and academic field trips (pkm)	Airplane (Long haul)	601,034	Intermunicipal bus: 19,515.1 pkm were due to staff trips and 41,568 pkm due to academic field trips	Transport, passengers, aircraft, long haul {CO}|
	Airplane (Medium haul)	77,084		Transport, passengers, aircraft, medium haul {CO}
	Airplane (Short haul)	105,798		Transport, passengers, aircraft, short haul {CO}
	Airplane (Very short haul)	536,536		Transport, passengers, aircraft, very short haul {CO}
	Intermunicipal bus	61,083.1		Transport, passenger coach {CO}| processing
Daily mobility (pkm)	Private car	7932,174.5	Private car and motorcycle: specific datasets were created according to the mix of kinds of vehicles owned by lecturers, administrative staff, and students	Transport, (lecturers, administrative, or students)'s vehicles mix {CO}
	Motorcycle	16,248,155.3		Transport, (lecturers, administrative, or students)'s motorcycles mix {CO
	Taxi or similar	630,640.5		Transport, taxi, or similar mix {CO}
	Urban bus	39,727,002.8		Transport, regular bus {CO}| market
Transport at the start/end of the semester (pkm)	Airplane (Medium haul)	10,133.8	Transport of students between their hometown and Pereira at the start and end of each semester	Transport, passengers, aircraft, medium haul {CO}
	Private car	10,029.8		Transport, students' vehicles mix {CO}
	Motorcycle	122,360		Transport, students' motorcycles mix {CO
	Intermunicipal bus	643,814.3		Transport, passenger coach {CO}| processing
Refrigerant air conditioning (kg)		1.8	41 air conditioning units with different capacities	Chlorodifluoromethane, HCFC-22, or R-22 {CO}| market
Extinguishing agents (kg)	ABC	1308.6	Amount of extinguishing agent recharged per year by type of extinguisher: ABC 1,240.6 kg; Satellite (ABC) 68 kg; BC 9.1 kg; CO_2_ 68.3 kg; H_2_O 4.5 kg; Solkaflam 93 kg	ABC extinguisher {CO}| mono ammonium phosphate, market
	BC	9.1		BC extinguisher {CO}| sodium bicarbonate, market
	CO_2_	68.3		CO_2_ extinguisher {CO}| carbon dioxide, market
	H_2_O	4.5		H_2_O extinguisher {CO}| tap water, market
	Solkaflam	93		Chlorodifluoromethane, HCFC-22, or R-22 {CO}| market
Ordinary solid waste (kg)		75,056.4	To sanitary landfill	Municipal solid waste {CO}| treatment of sanitary landfill
Chemical dangerous solid waste treatment (kg)		13,987.9	These wastes were treated in different locations and using techniques such as security landfill, lead exploitation, several exploitations, and incineration	0.907 tkm: Security landfill, Transport, lorry, unspecified {CO}|, EURO3
				0.206 tkm: Lead exploitation, Transport, lorry, unspecified {CO}|, EURO3
				0.201 tkm: Several exploitations, Transport, lorry, unspecified {CO}, EURO3
				Hazardous waste, for incineration {CO}| market for
Biological dangerous solid waste treatment (kg)		3928	The techniques used to treat these wastes were energy recovery and sterilization	Biological dangerous waste, autoclave sterilization treatment {CO}| market
				Biohazardous waste, for incineration {CO}| market

**Table 5:** Measured physicochemical characteristics of wastewater from the UTP campus

Parameter	Fine Arts WWTP(g/m^3^)			Sports WWTP(g/m^3^)
	Influent	Effluent	Influent	Effluent
BOD_5_	177.5	46.0	460.5	31.8
COD	395.5	115.5	971.0	78.8
Phosphorus (Total)	13.3	13.9	11.8	8.7
Orthophosphates	11.3	1.2	9.5	6.1
Nitrates	0.5	0.2	4.5	3.4
Nitrites	0.0	0.0	0.0	6.6
Ammoniacal nitrogen	129.0	130.0	105.0	83.2
Kjeldahl nitrogen NTK	220.0	149.0	146.5	107.5

Source: Average data from characterization results (GIAS, 2018a, 2018b)

**Table 6:** Transfer coefficients for WWTP with secondary treatment. Based on Doka (2009)

Parameter	Unit	Estimated quantity	Primary treatment	Secondary treatment		To water effluent
			To sludge	To sludge	To air	
BOD	g O_2_ /m^3^	Measured	39%	30.7%	22.3%	8.0%
COD	g O_2_ /m^3^	Measured)	32.0%	29.0%	21.0%	18.0%
DOC	g C /m^3^	TOC*0.68	0.0%	49.7%	36.0%	14.2%
TOC	g C /m^3^	BOD*0.65	32.0%	33.8%	24.5%	9.7%
Only the quantity of TOC to air is used for the estimation of carbon compounds emissions according to Table 8						
Total phosphorous	g P/m^3^	Measured	These primary and secondary treatments do not reduce phosphorous and phosphates			100%
Orthophosphates	g P-PO_4_ /m^3^	Measured				100%
Nitrates	g N-NO_3_ /m^3^	Measured	The fraction to sludge is in form of Particulate Nitrogen. It can be calculated from one of these parameters or from the TKN, using the coefficients in Table 4.18 (Doka, 2009) 'Part IV Wastewater treatment'			Measured
Nitrites	g N-NO_2_ /m^3^	Measured				Measured
Ammonia nitrogen	g N-NH_4_ /m^3^	Measured				Measured
Kjeldahll Nitrogen (TKN)	g /m^3^	Measured				-
Particulate Nitrogen^c^	g /m^3^		TKN*(22.3%)		TKN*(5.2%)	TKN*(1.9%)
SO_4_-S (dissolved)	g /m^3^	44	0%			100%
S particle	g /m^3^	2	100%			0
S total^**d**^	g /m^3^	46	4%			96%
Transfer coefficients of metals are based on Koppe and Stotzek, (1993) and Boller and Hafliger (1996)						
Cl (chloride)^a^	g /m^3^	30.03	0%			100%
F (Fluoride)^a^	g /m^3^	0.03277	0%			100%
As (Arsenic)	g /m^3^	0.0009	22%			78%
Cd (Cadmium)	g /m^3^	0.0002806	50%			50%
Co (Cobalt)	g /m^3^	0.001618	50%			50%
Cr (Chromium)^b^	g /m^3^	0.01223	50%			50%
Cu (Copper)	g /m^3^	0.03744	75%			25%
Hg (Mercury)	g /m^3^	0.0002	70%			30%
Mn (Manganese)	g /m^3^	0.053	50%			50%
Mo (Molybdenum) ^1^	g /m^3^	0.0009574	50%			50%
Ni (Nickel)	g /m^3^	0.006589	40%			60%
Pb (Lead)	g /m^3^	0.008631	90%			10%
Sn (Tin or Stannum)	g /m^3^	0.0034	59%			41%
Zn (Zinc)	g /m^3^	0.1094	70%			30%
Si (Silicon)^a^	g /m^3^	3.126	95%			5%
Fe (Iron)^a^	g /m^3^	7.093	50%			50%
Ca (Calcium)^a^	g /m^3^	50.83	10%			90%
Al (Aluminium)	g /m^3^	1.038	95%			5%
K (Potassium)^a^	g /m^3^	0.3989	0%			100%
Mg (Magnesium)^a^	g /m^3^	5.707	10%			90%
Na (Sodium)^a^	g /m^3^	2.186	0%			100%

^a^Transfer coefficients assumed in Ecoinvent report from other elements with similar characteristics

^b^Chromium emitted in the effluent is dissolved Cr^VI^, while chromium retained in the sludge is precipitated Cr^III^. Chromium in digested sludge and disposed in landfarming is inventoried as Cr^III^

^c^From the fraction of nitrogen emitted to air, 0.68% is emitted in form of N_2_O and 99.32% in form of elemental N_2_

^d^Sulphur to air form of SO_2_ and to water in form of SO_4_

**Table 7:** Transfer coefficients for WWTP from the digester and sludge treatment. Based on Doka (2009)

Parameter	Sludge digester		Emitted from the incineration of the fraction of digested sludge		
	To air	To digested sludge^1^	To air	To water	To groundwater^2^
BOD	12.8%	87.2%			6.98%
TOC	60.3%	39.7%	see Table 8	0.00101000%	0.76%
Particulate Nitrogen ^3^	60.3%	39.7%	see Table 8	0.10000000%	1.00%
S total ^4^	22.3%	77.7%	0.213%	7.14%	55.40%
Cl (chloride)	0	100.000000%	0.00108000%	90.90000000%	7.13%
F (Fluoride)	0	100.000000%	0.05000000%	5.60000000%	61.50%
As (Arsenic)	0.1300000%	99.870000%	0.00000102%	0.01000000%	55.00%
Cd (Cadmium)	0.0000450%	99.999955%	0.00551000%	0.04410000%	0.33%
Co (Cobalt)	0	100.000000%	0.00000318%	0.00100000%	85.00%
Cr (Chromium)	0	100.000000%	0.00000739%	0.31900000%	45.50%
Cu (Copper)	0	100.000000%	0.00073800%	0	80.10%
Hg (Mercury)	0.0002400%	99.999760%	0.00000345%	1.05000000%	0.57%
Mn (Manganese)	0	100.000000%	0.00000055%	0.00100000%	86.00%
Mo (Molybdenum)	0	100.000000%	0.20000000%	0	86.70%
Ni (Nickel)	0	100.000000%	0.00000432%	0	90.10%
Pb (Lead)	0.0000037%	99.999996%	0.00371000%	0.00186000%	6.64%
Sn (Tin or Stannum)	0.0000170%	99.999983%	0.13300000%	0.00133000%	49.60%
Zn (Zinc)	0	100.000000%	0.00163000%	0.01630000%	0.33%
Si (Silicon)	0	100.000000%	0.23300000%	0	91.90%
Fe (Iron)	0	100.000000%	0.00334000%	0.03340000%	89.90%
Ca (Calcium)	0	100.000000%	0.16700000%	0	86.20%
Al (Aluminium)	0	100.000000%	0.15600000%	0	85.30%
K (Potassium)	0	100.000000%	0.30100000%	0	66.80%
Mg (Magnesium)	0	100.000000%	0.13800000%	0	91.70%
Na (Sodium)	0	100.000000%	0.94100000%	0	61.40%

**Table 8:** Speciation of carbon (TOC) and nitrogen emissions transferred to the air

Speciation of carbon emissions (TOC) transferred to the air				
Compound	C-CO_2_	C–CO	C-NMVOC	C-CH_4_^a^
Fraction from TOC	98.30%	0.20%	0.0069%	1.40%
Stoichiometric ratio	3.6667	2.3333	1.25	1.3333
Speciation of nitrogen emissions transferred to the air				
Compound	N-NO_2_	N-NH_3_	N-N_2_O	N-N_2_^a^
Fraction from N	5.60%	1.70%	0.90%	91.80%
Stoichiometric ratio	3.2857	1.2143	3.1429	2

^a^When sludge is incinerated, the carbon fraction released in form of CH_4_ and the N fraction released in form of NO_2_ are reduced to 0.1523% and 4.06%, respectively due to the use of selective catalytic reduction (SCR) technologies to reduce nitrogen oxides (NOx)

**Table 9:** Direct uses of water at the Technological University of Pereira in 2017

Water inlets		Water outlets	
Denomination	Quantity	Denomination	Quantity
Drinking water	48,239 m^3^	Water for irrigation of gardens and other green areas	18,543.8 m^3^
		Untreated wastewater	10,408.2 m^3^
		WWTP Fine Arts	911.4 m^3^
		WWTP Sports	16,447 m^3^
		Evaporation in the WWTP	1928.6 m^3^
TOTAL	**48,239 m** ^**3**^	**TOTAL**	**48,239 m** ^**3**^

**Table 10:** Wastewater treatment inventories per m^3^ on the UTP campus – emissions to air

Output	Unit	Fine Arts WWTP	Sports WWTP	Without treatment
Aluminum	kg	0.00E + 00	0.00E + 00	0.00E + 00
Ammonia	kg	6.71E − 03	4.47E − 03	0.00E + 00
Arsenic	kg	2.57E − 10	2.57E − 10	0.00E + 00
Cadmium	kg	6.31E − 14	6.31E − 14	0.00E + 00
Calcium	kg	0.00E + 00	0.00E + 00	0.00E + 00
Carbon dioxide, biogenic	kg	8.65E − 01	5.97E − 01	0.00E + 00
Carbon monoxide, biogenic	kg	6.92E − 04	4.78E − 04	0.00E + 00
Chromium	kg	0.00E + 00	0.00E + 00	0.00E + 00
Cobalt	kg	0.00E + 00	0.00E + 00	0.00E + 00
Copper	kg	0.00E + 00	0.00E + 00	0.00E + 00
Dinitrogen monoxide	kg	9.67E − 04	6.44E − 04	0.00E + 00
Iron	kg	0.00E + 00	0.00E + 00	0.00E + 00
Lead	kg	2.87E − 13	2.87E − 13	0.00E + 00
Magnesium	kg	0.00E + 00	0.00E + 00	0.00E + 00
Manganese	kg	0.00E + 00	0.00E + 00	0.00E + 00
Mercury	kg	3.36E − 13	3.36E − 13	0.00E + 00
Methane, biogenic	kg	2.77E − 03	1.91E − 03	0.00E + 00
Molybdenum	kg	0.00E + 00	0.00E + 00	0.00E + 00
Nickel	kg	0.00E + 00	0.00E + 00	0.00E + 00
Nitrogen oxides	kg	5.44E − 03	3.62E − 03	0.00E + 00
NMVOC	kg	1.28E − 05	8.83E − 06	0.00E + 00
Phosphorus	kg	0	0	0
Silicon	kg	0.00E + 00	0.00E + 00	0.00E + 00
Sulfur dioxide	kg	8.82E − 04	8.82E − 04	0.00E + 00
Tin	kg	3.41E − 13	3.41E − 13	0.00E + 00
Water	m^3^	0.1	0.1	0
Zinc	kg	0.00E + 00	0.00E + 00	0.00E + 00

**Table 11:** Wastewater treatment inventories per m^3^ on the UTP campus – emissions to water (river)

Output	Unit	Fine Arts WWTP	Sports WWTP	Without treatment
Aluminum	kg	5.19E − 05	5.19E − 05	1.04E − 03
Ammonium, ion	kg	1.30E − 01	8.32E − 02	1.29E − 01
Arsenic	kg	7.02E − 07	7.02E − 07	9.00E − 07
BOD5	kg	4.60E − 02	3.18E − 02	1.78E − 01
Cadmium	kg	1.40E − 07	1.40E − 07	2.81E − 07
Calcium	kg	4.57E − 02	4.57E − 02	5.08E − 02
Chloride	kg	3.00E − 02	3.00E − 02	3.00E − 02
Chromium VI	kg	6.12E − 06	6.12E − 06	1.22E − 05
Cobalt	kg	8.09E − 07	8.09E − 07	1.62E − 06
COD	kg	1.16E − 01	7.88E − 02	3.96E − 01
Copper	kg	9.36E − 06	9.36E − 06	3.74E − 05
DOC	kg	3.62E − 02	2.50E − 02	7.85E − 02
Fluoride	kg	3.28E − 05	3.28E − 05	3.28E − 05
Iron	kg	3.55E − 03	3.55E − 03	7.09E − 03
Lead	kg	8.63E − 07	8.63E − 07	8.63E − 06
Magnesium	kg	5.14E − 03	5.14E − 03	5.71E − 03
Manganese	kg	2.65E − 05	2.65E − 05	5.30E − 05
Mercury	kg	6.00E − 08	6.00E − 08	2.00E − 07
Molybdenum	kg	4.79E − 07	4.79E − 07	9.57E − 07
Nickel	kg	3.95E − 06	3.95E − 06	6.59E − 06
Nitrate	kg	1.80E − 04	3.45E − 03	5.39E − 04
Nitrite	kg	5.00E − 06	6.61E − 03	5.00E − 06
Nitrogen, atmospheric	kg	4.18E − 03	2.78E − 03	2.57E − 02
Phosphate	kg	1.13E − 02	9.51E − 03	1.13E − 02
Potassium	kg	3.99E − 04	3.99E − 04	3.99E − 04
Silicon	kg	1.56E − 04	1.56E − 04	3.13E − 03
Sodium	kg	2.19E − 03	2.19E − 03	2.19E − 03
Sulfate	kg	1.32E − 01	1.32E − 01	1.38E − 01
Tin	kg	1.39E − 06	1.39E − 06	3.40E − 06
TOC	kg	3.62E − 02	2.50E − 02	1.15E − 01
Water	m^3^	0.9	0.9	1
Zinc	kg	3.28E − 05	3.28E − 05	1.09E − 04

**Table 12:** Wastewater treatment inventories per m^3^ on the UTP campus – emissions to soil (agriculture)

Output	Unit	Fine Arts WWTP	Sports WWTP	Without treatment
Aluminum	kg	9.86E − 04	9.86E − 04	0.00E + 00
Arsenic	kg	1.98E − 07	1.98E − 07	0.00E + 00
Cadmium	kg	1.40E − 07	1.40E − 07	0.00E + 00
Calcium	kg	5.08E − 03	5.08E − 03	0.00E + 00
Carbon	kg	9.76E − 02	6.74E − 02	0.00E + 00
Chromium	kg	6.12E − 06	6.12E − 06	0.00E + 00
Cobalt	kg	8.09E − 07	8.09E − 07	0.00E + 00
Copper	kg	2.81E − 05	2.81E − 05	0.00E + 00
Iron	kg	3.55E − 03	3.55E − 03	0.00E + 00
Lead	kg	7.77E − 06	7.77E − 06	0.00E + 00
Magnesium	kg	5.71E − 04	5.71E − 04	0.00E + 00
Manganese	kg	2.65E − 05	2.65E − 05	0.00E + 00
Mercury	kg	1.40E − 07	1.40E − 07	0.00E + 00
Molybdenum	kg	4.79E − 07	4.79E − 07	0.00E + 00
Nickel	kg	2.64E − 06	2.64E − 06	0.00E + 00
Silicon	kg	2.97E − 03	2.97E − 03	0.00E + 00
Sulfur	kg	1.54E − 03	1.54E − 03	0.00E + 00
Tin	kg	2.01E − 06	2.01E − 06	0.00E + 00
Zinc	kg	7.66E − 05	7.66E − 05	0.00E + 00

**Table 13:** Summary results of water footprint due to scarcity—water consumption

Input or activity	m^3^	Contribution
Irrigation water consumption	18,543.8	38.4%
Wastewater Sports WWTP	18,274.4	37.9%
Wastewater Fine Arts WWTP	1012.6	2.1%
Untreated wastewater	10,408.2	21.6%
Direct activities	**48,239**	**13.5%**
Drinking water consumption from the aqueduct network	78.4	0.0%
Electricity consumption	9267.2	3%
Fuel consumption	113.2	0.0%
Paper consumption	597.8	0.2%
Infrastructure	296,913	95.9%
Staff trips	200	0.1%
Academic field trips	117.3	0.0%
Student journeys beginning/end of the semester	294.2	0.1%
Daily mobility university community	1985.5	0.6%
Refrigerant recharge Air conditioning	0.2	0.0%
Extinguisher recharge	109.8	0.0%
Solid waste disposal in landfill	25.0	0.0%
Chemical dangerous solid waste management	27	0.0%
Biological dangerous solid waste management	4.4	0.0%
Indirect activities	**309,732.7**	**86.5%**
Total m^3^	**357,971.7**	**100.0%**

**Table 14:** Contribution of activities to midpoint water quality impacts

Input or activity	Freshwater eutrophication	Marine eutrophication	Freshwater ecotoxicity	Marine ecotoxicity
Irrigation water consumption	0.0%	0.0%	0.0%	0.0%
Wastewater Sports WWTP	57.4%	51.8%	34.9%	34.6%
Wastewater Fine Arts WWTP	3.8%	4.3%	1.9%	1.9%
Untreated wastewater	38.8%	43.8%	63.2%	63.5%
Direct impact	**1.2%**	**43.9%**	**0.0%**	**0.0%**
Drinking water consumption from the aqueduct network	0.0%	0.0%	0.0%	0.0%
Electricity consumption	2%	1.1%	0.1%	4.7%
Fuel consumption	0.0%	0.1%	0.0%	0.0%
Paper consumption	0.0%	0.2%	0.0%	0.0%
Infrastructure	97.3%	90.5%	99.9%	92.4%
Staff trips	0.1%	0.1%	0.0%	0.1%
Academic field trips	0.0%	0.1%	0.0%	0.0%
Student journeys beginning/end of the semester	0.1%	0.1%	0.0%	0.1%
Daily mobility university community	0.5%	0.9%	0.0%	0.9%
Refrigerant recharge Air conditioning	0.0%	0.0%	0.0%	0.0%
Extinguisher recharge	0.0%	0.0%	0.0%	0.0%
Solid waste disposal in landfill	0.0%	6.8%	0.0%	1.8%
Chemical dangerous solid waste management	0.0%	0.0%	0.0%	0.0%
Biological dangerous solid waste management	0.0%	0.0%	0.0%	0.0%
Indirect impact	**98.8%**	**56.1%**	**100.0%**	**100.0%**
Total kg	**8.3 (P eq)**	**1.6 (N eq)**	**130.2 × 10** ^**6**^ ** (1,4 DCB)**	**2.42 × 10** ^**6**^ ** (1,4 DCB)**
